# The 100 most-cited articles in hypothermic brain protection journals: a bibliometric and visualized analysis

**DOI:** 10.3389/fneur.2024.1433025

**Published:** 2024-11-05

**Authors:** Liren Hu, Sirui Geli, Feiyu Long, Liang Nie, Jiali Wu, Jun Zhou, Maohua Wang, Yingxu Chen

**Affiliations:** ^1^Department of Anesthesiology, The Affiliated Hospital, Southwest Medical University, Luzhou, Sichuan Province, China; ^2^Anesthesiology and Critical Care Medicine Key Laboratory of Luzhou, The Affiliated Hospital, Southwest Medical University, Sichuan Province, China; ^3^Department of Anesthesiology, Fushun County People’s Hospital, Zigong, Sichuan Province, China

**Keywords:** hypothermia, brain protection, surgery, bibliometric analysis, citations highlights

## Abstract

**Introduction:**

A bibliometric analysis is used to assess the impact of research in a particular field. However, a specialized bibliometric analysis focused on hypothermic brain protection has not yet been conducted. This study aimed to identify the 100 most-cited articles published in the field of hypothermic brain protection and analyze their bibliometric characteristics.

**Methods:**

After screening articles from the Web of Science citation database, complete bibliographic records were imported into Python for data extraction. The following parameters were analyzed: title, author’s name and affiliation, country, publication year, publication date, first author, corresponding author, study design, language, number of citations, journal impact factors, keywords, Keywords Plus^®^, and research topic.

**Results:**

The 100 articles were published between 1990 and 2016. The citation frequency for each publication ranged from 86 to 470. Among the 100 articles, 73 were original articles, 18 were review articles, 8 were clinical articles, and 1 was editorial material. These papers were published in 37 journals, with the *Journal of Cerebral Blood Flow and Metabolism* being the most prolific with 15 papers. Eighteen countries contributed to the 100 publications, 51 of which were from United States institutions. In addition, the keywords in the Sankey plot indicated that research in the field of hypothermic brain protection is growing deeper and overlapping with other disciplines.

**Discussion:**

The results provide an overview of research on hypothermic brain protection, which may help researchers better understand classical research, historical developments, and new discoveries, as well as providing ideas for future research.

## Highlights

- The unique advantage of this study is that it is the first bibliometric study to identify and characterize articles in the field of hypothermic brain protection in all journals of the Science Network (SCIE).
- Most bibliometric studies exclude nonprofessional journals.
- We generated a more comprehensive list of the 100 most-cited articles in the field of hypothermic brain protection by including all journals in the analysis.

## Introduction

Hypothermic brain protection is an important technique used in neurosurgery to mitigate ischemic and hypoxic injuries. Moreover, lowering the temperature of the brain tissue can protect neurological function (1). Recent research has focused on understanding the mechanisms of hypothermic brain protection and its ability to protect brain functions (2). Hypothermia inhibits the generation of free radicals, reduces apoptosis, and stabilizes cell membrane integrity (3). Clinical methods for hypothermic brain protection include surface, nasopharyngeal, and intravenous cooling (4). However, hypothermia can also lead to complications, such as immunosuppression and renal failure, highlighting the need for further investigation to determine the optimal hypothermic brain protection protocol (5).

Several related studies in the field of hypothermic brain protection have been published in various journals. Analysis of these articles is crucial to evaluate their impact on basic research, clinical practice, and the surgical profession. Bibliometric analysis is an excellent method to assess this impact (6).

Bibliometrics is a discipline that uses quantitative and statistical methods to analyze scientific literature (7). It provides valuable information and reveals the laws and trends in the development of a particular scientific discipline (8). In neurological surgery, bibliometric analysis has been widely applied to analyze research hotspots and developmental trajectories of various diseases, including cerebral aneurysms, stroke, and traumatic brain injury (9–11). This analysis provides guidance for future research directions and resource allocation in the field of neurosurgery. However, specialized bibliometric analyses have not yet been performed in the field of hypothermic brain protection.

Therefore, this study aimed to identify the 100 most-cited articles published in the field of hypothermic brain protection using bibliometric methods. We analyzed their bibliometric characteristics to provide insights into the developments in this field.

## Materials and methods

### Ethical considerations

This study analyzed and described previously published articles; therefore, no ethical approval was required.

### Data sources and search strategies

The Clarivate Analytics’ Web of Science (WOS) (1980–present) citation database was used as the data source to identify articles in the field of hypothermic brain protection and track their citations. Considering the broad range of topics covered in the articles on hypothermia-related brain protection, we conducted a pre-search to determine the best search formula. The last search was conducted on August 21, 2023, using the expressions detailed in Supplementary Table S1. All obtained references, including bibliographic and citation data, were exported from the database and subsequently imported into document management software (Zotero, 6.0.30; https://www.zotero.org/) to remove duplications and screen them. The search strategy produced 1,847 records, listed in descending order, based on the number of citations retrieved from the source database.

### Study selection and data extraction

Two independent researchers (Geli and Huang) screened the literature in the database in descending order based on WOS citations. Literature in the field of non-hypothermic brain protection was excluded based on the title and abstract. For uncertain articles, the full text was obtained for accurate inclusion or exclusion determination. During the study selection process, discrepancies between investigators were resolved by a third investigator (Hu). The evaluation was terminated at the 100th paper with the highest number of citations. Finally, the 100 most-cited articles in the field of hypothermic brain protection were listed for further analysis. Complete bibliographic records were exported from the WOS in plain text or Excel (Microsoft Corporation, Redmond, WA, United States) format and imported into Python (version 3.11.5; https://www.python.org/downloads/release/python-3115/) for data extraction.

The following information was extracted using Python and stored in Microsoft Excel (Microsoft Corporation): article title, author, abstract, keywords, year of publication, published journal, cited references, PMID, DOI, total citations, and annual average citations. We also determined the impact factor (IF) of the articles based on the currently published journal citation report (JCR^®^ IF 2023).

### Data analysis and visualization

**Figure 1:**
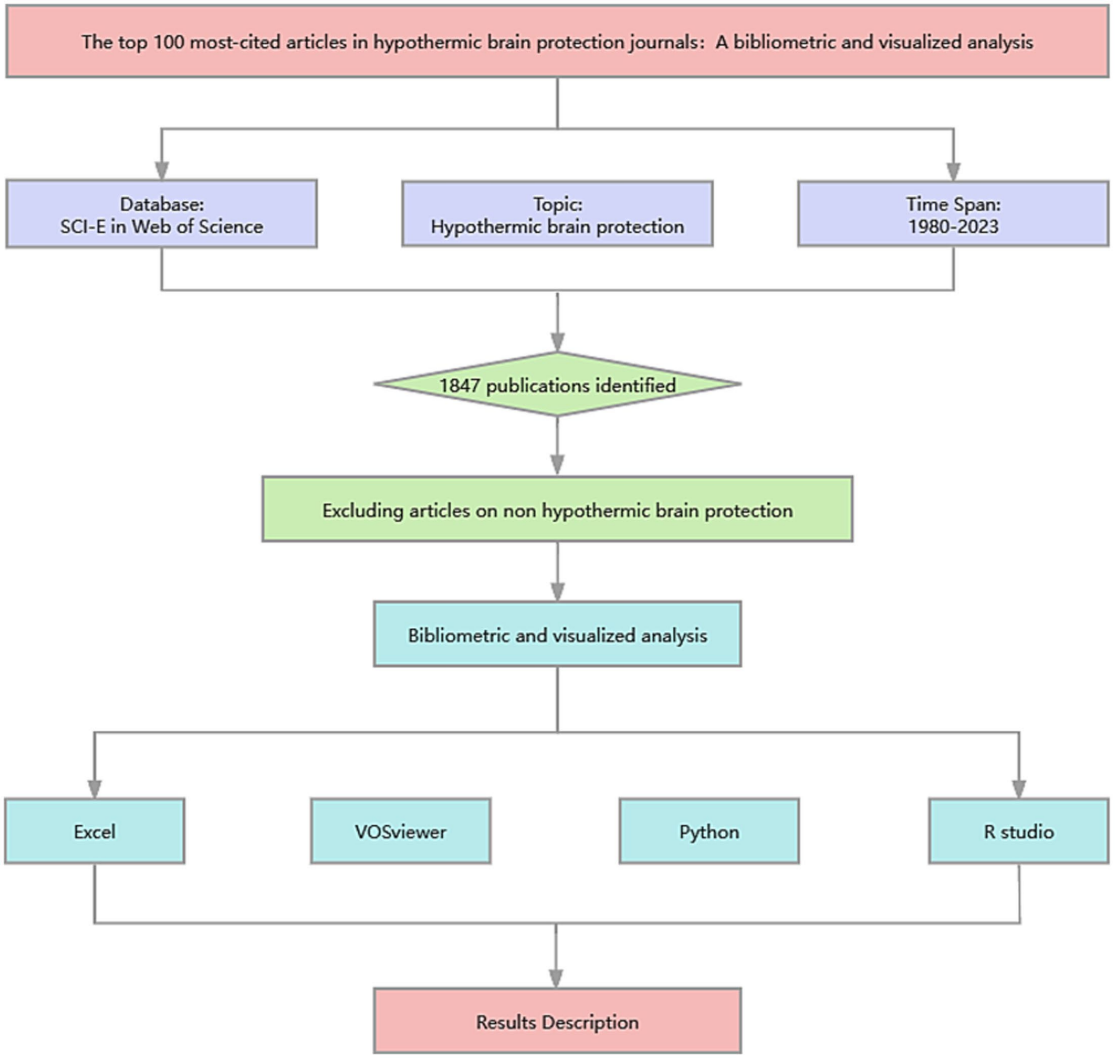
Study flow diagram created using ProcessOn.com. The Web of Science (WOS) (1980–present) citation database was used to identify articles in the field of hypothermic brain protection. The search strategy yielded 1,847 records, with the final selection of 100 articles made by the researchers. Data were then standardized and imported into Excel, VOSviewer, Python, and R Studio for bibliometric and visual analyses.

Descriptive statistics of the selected articles were analyzed using Microsoft Excel 2023 (Microsoft Corporation), Python (version 3.11.5), and VOSviewer [version 1.6.20; developed by van Eck and Waltman (12)], a literature knowledge visualization software for constructing a bibliometric network. Before data analysis, the obtained data were standardized. All authors were checked through their institutions or email addresses to eliminate potential confusion from authors sharing the same names and initials, ensuring that they were specific individuals. The names of all institutions, such as universities and research centers, were reviewed and included at the same level, whereas the individual departments or research units under them were removed. Articles from Northern Ireland and Britain were considered to be from the United Kingdom. Different keywords with the same meaning were merged into one term (e.g., head injury and brain injury were collectively classified as brain injury). Some authors in the top 20 articles did not provide keywords (*n* = 7), and three independent researchers discussed and determined the keywords for these articles based on Keywords Plus^®^. All references were standardized to create a unified list. Excel (Microsoft Corporation) was used to list the basic characteristics of the selected documents in a tabular form. The bibliometric network was graphically generated using the VOSviewer software (12). In a network visualization map, different nodes represent different elements such as countries, institutions, authors, or terms. The links between nodes represent relationships such as coauthors, co-citations, or co-occurrences, and are weighted by the total link strength (13). Python (version 3.11.5) and R (version 4.3.1; R Foundation for Statistical Computing, Vienna, Austria) were used to construct radar charts describing article types and publication years and a Sankey plot was created to describe the relationships among authors, countries, and keywords. The packages used in Python and R are listed in Supplementary Table S2. The research process is shown in Figure 1.

## Results

### Citations

The 100 most-cited articles from all journals were published between 1990 and 2016, as shown in Table 1, and are listed in descending order based on the total WOS citation count on the search day (1, 14–112). Additionally, the table includes the year of publication, journal name, IF, first author, corresponding author, and average citations per year count based on WOS data.

**Table 1:** List of the 100 most-cited articles in hypothermic brain protection journals.

Ranking	Title	JCR^®^ IF	Total citations	Average citation	Journal	First author	Corresponding author	Published year
1	Glutamate release and free-radical production following brain injury—effects of posttraumatic hypothermia	4.7	470	16.8	Journal of Neurochemistry	Globus, MYT	Globus, MYT	1995
2	Marked protection by moderate hypothermia after experimental traumatic brain injury	6.3	440	13.8	Journal of Cerebral Blood Flow and Metabolism	Clifton, GL	Dr. G. L. Clifton	1991
3	Delay in cooling negates the beneficial effect of mild resuscitative cerebral hypothermia after cardiac-arrest in dogs— a prospective, randomized study	8.8	417	13.9	Critical Care Medicine	Kuboyama, K	Peter Safar	1993
4	Therapeutic modulation of brain temperature—relevance to ischemic brain injury	6.9	416	13.4	Cerebrovascular and Brain Metabolism Reviews	Ginsberg, MD	Ginsberg, MD	1992
5	Intraischemic but not postischemic brain hypothermia protects chronically following global forebrain ischemia in rats	6.3	410	13.7	Journal of Cerebral Blood Flow and Metabolism	Dietrich, WD	Dietrich, WD	1993
6	Delayed postischemic hypothermia—a 6 month survival study using behavioral and histological assessments of neuroprotection	5.3	409	14.6	Journal of Neuroscience	Colbourne, F	Frederick Colbourne	1995
7	Mild intraoperative hypothermia during surgery for intracranial aneurysm	158.5	345	19.2	New England Journal of Medicine	Todd, MM	Todd, MM	2005
8	Effects of hypothermia on energy metabolism in mammalian central nervous system	6.3	328	16.4	Journal of Cerebral Blood Flow and Metabolism	Erecinska, M	Erecinska, M	2003
9	Protective effects of moderate hypothermia after neonatal hypoxia-ischemia: short-and long-term outcome	3.6	273	10.9	Pediatric Research	Bona, E	Thoresen, M	1998
10	Posttraumatic brain hypothermia reduces histopathological damage following concussive brain injury in the rat	12.7	271	9.3	Acta Neuropathologica	Dietrich, WD	Dietrich, WD	1994
11	The effect of hypothermic cardiopulmonary bypass and total circulatory arrest on cerebral metabolism in neonates, infants, and children	6	258	8.1	Journal of Thoracic and Cardiovascular Surgery	Greeley, WJ	Greeley, WJ	1991
12	Brain temperature alters hydroxyl radical production during cerebral ischemia reperfusion in rats	6.3	248	9.2	Journal of Cerebral Blood Flow and Metabolism	Kil, HY	Dr. C. A. Piantadosi	1996
13	Environment-, drug- and stress-induced alterations in body temperature affect the neurotoxicity of substituted amphetamines in the C57BL/6J mouse	3.5	237	8.2	Journal of Pharmacology and Experimental Therapeutics	Miller, DB	Miller, DB	1994
14	Induced hypothermia in critical care medicine: a review	8.8	233	11.7	Critical Care Medicine	Bernard, SA	Bernard, SA	2003
15	Antegrade cerebral perfusion with cold blood: a 13-year experience	4.6	228	9.5	Annals of Thoracic Surgery	Bachet, J	Bachet, J	1999
16	Treatment advances in neonatal neuroprotection and neurointensive care	48	219	18.3	Lancet Neurology	Johnston, Michael V	Johnston, MV	2011
17	The relationship among canine brain temperature, metabolism, and function during hypothermia	8.8	213	6.7	Anesthesiology	Michenfelder, JD	Michenfelder, JD	1991
18	Xenon and hypothermia combine to provide neuroprotection from neonatal asphyxia	11.2	213	11.8	Annals of Neurology	Ma, DQ	Maze, M	2005
19	Low environmental temperatures or pharmacological agents that produce hypothermia decrease methamphetamine neurotoxicity in mice	2.9	185	6.4	Brain Research	Ali, SF	Ali, SF	1994
20	Whole-body hypothermia for neonatal encephalopathy: animal observations as a basis for a randomized, controlled pilot study in term infants	8	180	8.6	Pediatrics	Shankaran, S	Shankaran, S	2002
21	Moderate hypothermia mitigates neuronal damage in the rat-brain when initiated several hours following transient cerebral-ischemia	12.7	177	6.1	Acta Neuropathologica	Coimbra, C	C. Coimbra	1994
22	Mild hypothermia reduces apoptosis of mouse neurons *in vitro* early in the cascade	6.3	176	8.4	Journal of Cerebral Blood Flow and Metabolism	Xu, LJ	Giffard, RG	2002
23	Long-lasting neuroprotective effect of postischemic hypothermia and treatment with an anti-inflammatory/antipyretic drug—evidence for chronic encephalopathic processes following ischemia	8.3	176	6.5	Stroke	Coimbra, C	Coimbra, C	1996
24	Posthypoxic cooling of neonatal rats provides protection against brain injury	4.4	174	6.4	Archives of Disease in Childhood-fetal and Neonatal Edition	Thoresen, M	Dr. Marianne Thoresen	1996
25	Xenon and hypothermia combine additively, offering long-term functional and histopathologic neuroprotection after neonatal hypoxia/ischemia	8.3	174	11.6	Stroke	Hobbs, Catherine	Thoresen, M	2008
26	Chronic histopathological consequences of fluid-percussion brain injury in rats: effects of post-traumatic hypothermia	12.7	169	6.5	Acta Neuropathologica	Bramlett, HM	Bramlett, HM	1997
27	Effect of hypothermia on cerebral blood flow and metabolism in the pig	4.6	168	8.0	Annals of Thoracic Surgery	Ehrlich, MP	Griepp, RB	2002
28	Indefatigable ca1 sector neuroprotection with mild hypothermia induced 6 h after severe forebrain ischemia in rats	6.3	163	6.8	Journal of Cerebral Blood Flow and Metabolism	Colbourne, F	Colbourne, F	1999
29	Brain injury following trial of hypothermia for neonatal hypoxic-ischaemic encephalopathy	4.9	161	14.6	Archives of Disease in Childhood-Fetal and Neonatal Edition	Shankaran, Seetha	Shankaran, S	2012
30	Protective effects of brain hypothermia on behavior and histopathology following global cerebral-ischemia in rats	2.9	158	5.1	Brain Research	Green, EJ	Green, EJ	1992
31	RBM3 mediates structural plasticity and protective effects of cooling in neurodegeneration	64.8	155	19.4	Nature	Peretti, Diego	Mallucci, GR	2015
32	Hypothermia prevents ischemia-induced increases in hippocampal glycine concentrations in rabbits	8.3	152	4.8	Stroke	Baker, AJ	Dr. M. H. Zornow	1991
33	Influence of mild hypothermia on inducible nitric oxide synthase expression and reactive nitrogen production in experimental stroke and inflammation	5.3	148	7.0	Journal of Neuroscience	Han, HS	Yenari, MA	2002
34	Mild hypothermia inhibits inflammation after experimental stroke and brain inflammation	8.3	146	7.3	Stroke	Deng, H	Yenari, MA	2003
35	General versus specific actions of mild-moderate hypothermia in attenuating cerebral ischemic damage	6.3	145	9.1	Journal of Cerebral Blood Flow and Metabolism	Zhao, Heng	Zhao, H	2007
36	The importance of brain temperature in cerebral injury	4.2	143	4.6	Journal of Neurotrauma	Dietrich, WD	Dietrich, WD	1992
37	Protection against hippocampal ca1 cell loss by postischemic hypothermia is dependent on delay of initiation and duration	3.6	142	4.6	Metabolic Brain Disease	Carroll, M	Carroll, M	1992
38	Co-administration of MDMA with drugs that protect against MDMA neurotoxicity produces different effects on body temperature in the rat	3.5	142	5.3	Journal of Pharmacology and Experimental Therapeutics	Malberg, JE	Karen Sabol	1996
39	Twenty-four hours of mild hypothermia in unsedated newborn pigs starting after a severe global hypoxic-ischemic insult is not neuroprotective	3.6	138	6.3	Pediatric Research	Thoresen, M	Thoresen, M	2001
40	Posttraumatic brain hypothermia provides protection from sensorimotor and cognitive-behavioral deficits	4.2	135	4.8	Journal of Neurotrauma	Bramlett, HM	Edward J. Green	1995
41	Hypothermia for acute brain injury-mechanisms and practical aspects	38.1	134	12.2	Nature Reviews Neurology	Choi, H. Alex	Mayer, SA	2012
42	Hypothermia as a potential treatment for cerebral-ischemia		131	4.4	Cerebrovascular and Brain Metabolism Reviews	Maher, J		1993
43	Systemic inflammatory challenges compromise survival after experimental stroke via augmenting brain inflammation, blood-brain barrier damage and brain oedema independently of infarct size	9.3	130	10.8	Journal Of Neuroinflammation	Denes, Adam	Denes, A	2011
44	Hibernation in ground squirrels induces state and species-specific tolerance to hypoxia and aglycemia: an *in vitro* study in hippocampal slices	6.3	128	5.1	Journal of Cerebral Blood Flow and Metabolism	Frerichs, KU	Frerichs, KU	1998
45	Hypothermia in the management of traumatic brain injury—a systematic review and meta-analysis	38.9	127	6.4	Intensive Care Medicine	Henderson, WR	Henderson, WR	2003
46	Protection in animal models of brain and spinal cord injury with mild to moderate hypothermia	4.2	125	8.9	Journal of Neurotrauma	Dietrich, W. Dalton	Dietrich, WD	2009
47	A comfortable hypothesis reevaluated—cerebral metabolic depression and brain protection during ischemia	8.8	125	4.0	Anesthesiology	Todd, MM	Todd, MM	1992
48	Delayed induction of mild hypothermia to reduce infarct volume after temporary middle cerebral-artery occlusion in rats	4.1	125	4.3	Journal of Neurosurgery	Karibe, H	Philip R. Weinstein	1994
49	Neuroprotection after several days of mild, drug-induced hypothermia	6.3	124	4.6	Journal of Cerebral Blood Flow and Metabolism	Nurse, S	Nurse, S	1996
50	Selective antegrade cerebral perfusion and mild (28°C–30°C) systemic hypothermic circulatory arrest for aortic arch replacement: results from 1002 patients	6	124	11.3	Journal of Thoracic and Cardiovascular Surgery	Zierer, Andreas	Zierer, A	2012
51	Effects of isoflurane and hypothermia on glutamate receptor-mediated calcium influx in brain-slices	8.8	123	4.2	Anesthesiology	Bickler, PE	Bickler, PE	1994
52	Cirp protects against tumor necrosis factor-alpha-induced apoptosis via activation of extracellular signal-regulated kinase	5.1	123	7.2	Biochimica et Biophysica Acta-Molecular Cell Research	Sakurai, Toshiharu	Fujita, J	2006
53	Neuroprotective adaptations in hibernation: therapeutic implications for ischemia-reperfusion, traumatic brain injury and neurodegenerative diseases	7.4	122	5.5	Free Radical Biology and Medicine	Drew, KL	Drew, KL	2001
54	A comparison of the cerebral protective effects of isoflurane and mild hypothermia in a model of incomplete forebrain ischemia in the rat	8.8	122	3.9	Anesthesiology	Sano, T	Drummond	1992
55	Therapeutic hypothermia: neuroprotective mechanisms	3.1	121	7.6	Frontiers in Bioscience-Landmark	Liu, Liping	Yenari, MA	2007
56	Temperature modulation of ischemic neuronal death and inhibition of calcium calmodulin-dependent protein kinase-ii in gerbils	8.3	120	3.6	Stroke	Churn, SB	Robert J. DeLorenzo	1990
57	Resuscitative hypothermia	8.8	119	4.4	Critical Care Medicine	Marion, DW	Marion, DW	1996
58	Mild-to-moderate hypothermia in aortic arch surgery using circulatory arrest: a change of paradigm?	3.4	118	10.7	European Journal of Cardio-Thoracic Surgery	Urbanski, Paul P	Urbanski, PP	2012
59	Persistent neuroprotection with prolonged postischemic hypothermia in adult rats subjected to transient middle cerebral artery occlusion	5.3	118	5.1	Experimental Neurology	Corbett, D	Colbourne, F	2000
60	Metabolic downregulation—a key to successful neuroprotection?	8.3	118	7.9	Stroke	Yenari, Midori	Yenari, M	2008
61	Cooling combined with immediate or delayed xenon inhalation provides equivalent long-term neuroprotection after neonatal hypoxia-ischemia	6.3	118	8.4	Journal of Cerebral Blood Flow and Metabolism	Thoresen, Marianne	Dingley, J	2009
62	Antegrade selective cerebral perfusion in thoracic aorta surgery: safety of moderate hypothermia	3.1	117	7.3	European Journal of Cardio-Thoracic Surgery	Pacini, Davide	Pacini, D	2007
63	Occurrence of potentially detrimental temperature alterations in hospitalized patients at risk for brain injury	8.9	117	4.7	Mayo Clinic Proceedings	Albrecht, RF	Lanier, WL	1998
64	Influence of hypothermia on post-ischemic inflammation: role of nuclear factor kappa B (NFkappaB)	4.2	116	6.8	Neurochemistry International	Yenari, Midori A	Yenari, MA	2006
65	Mild posttraumatic hypothermia reduces mortality after severe controlled cortical impact in rats	6.3	115	4.3	Journal of Cerebral Blood Flow and Metabolism	Clark, RSB	P. M. Kochanek	1996
66	Topiramate extends the therapeutic window for hypothermia-mediated neuroprotection after stroke in neonatal rats	8.3	115	6.1	Stroke	Liu, YQ	Silverstein, FS	2004
67	Low-flow hypothermic cardiopulmonary bypass protects the brain	6	114	3.6	Journal of Thoracic and Cardiovascular Surgery	Swain, JA	Julie Swain	1991
68	Posttraumatic hypothermia in the treatment of axonal damage in an animal model of traumatic axonal injury	4.1	112	4.5	Journal of Neurosurgery	Koizumi, H	Povlishock, JT	1998
69	Regulation of therapeutic hypothermia on inflammatory cytokines, microglia polarization, migration and functional recovery after ischemic stroke in mice	6.1	110	15.7	Neurobiology of Disease	Lee, Jin Hwan	Yu, SP	2016
70	Cooling the injured brain: how does moderate hypothermia influence the pathophysiology of traumatic brain injury	3.1	109	6.8	Current Pharmaceutical Design	Sahuquillo, Juan	Sahuquillo, J	2007
71	A study of brain protection during total arch replacement comparing antegrade cerebral perfusion versus hypothermic circulatory arrest, with or without retrograde cerebral perfusion: analysis based on the japan adult cardiovascular surgery database	6	108	13.5	Journal of Thoracic and Cardiovascular Surgery	Okita, Yutaka	Okita, Y	2015
72	Effects of pH on brain energetics after hypothermic circulatory arrest	4.6	107	3.6	Annals of Thoracic Surgery	Aoki, M	Dr. Jonas	1993
73	Postischemic hypothermia and il-10 treatment provide long-lasting neuroprotection of ca1 hippocampus following transient global ischemia in rats	5.3	106	4.4	Experimental Neurology	Dietrich, WD	Dietrich, WD	1999
74	Moderate posttraumatic hypothermia decreases early calpain-mediated proteolysis and concomitant cytoskeletal compromise in traumatic axonal injury	5.3	102	4.3	Experimental Neurology	Buki, A	Buki, A	1999
75	Protective effects of moderate hypothermia on behavioral deficits but not necrotic cavitation following cortical impact injury in the rat	4.2	101	4.0	Journal of Neurotrauma	Dixon, CE	Hayes, RL	1998
76	Mild hypothermia attenuates cytochrome c release but does not alter Bcl-2 expression or caspase activation after experimental stroke	6.3	100	4.8	Journal of Cerebral Blood Flow and Metabolism	Yenari, MA	Yenari, MA	2002
77	Moderate hypothermia and unilateral selective antegrade cerebral perfusion: a contemporary cerebral protection strategy for aortic arch surgery	4.6	100	7.7	Annals of Thoracic Surgery	Leshnower, Bradley G	Chen, EP	2010
78	Prolonged mild hypothermia therapy protects the brain against permanent focal ischemia	8.3	98	4.5	STROKE	Yanamoto, H	Yanamoto, H	2001
79	Role of hypothermia in the mechanism of protection against serotonergic toxicity. II. Experiments with methamphetamine, p-chloroamphetamine, fenfluramine, dizocilpine and dextromethorphan	3.5	98	3.5	Journal of Pharmacology and Experimental Therapeutics	Farfel, GM	Lewis Seiden	1995
80	Intracerebral temperature in neurosurgical patients	4.8	98	3.1	Neurosurgery	Mellergard, P	Mellergard, P	1991
81	Therapeutic hypothermia for acute stroke	48	96	4.8	Lancet Neurology	Olsen, TS	Olsen, TS	2003
82	Cerebral oxygen-metabolism during hypothermic circulatory arrest in humans	4.1	96	3.2	Journal of Neurosurgery	Ausman, JI	Ausman, JI	1993
83	Prospective randomized trial of normothermic versus hypothermic cardiopulmonary bypass on cognitive function after coronary artery bypass graft surgery	8.8	95	4.3	Anesthesiology	Grigore, AM	Newman, MF	2001
84	Delayed, spontaneous hypothermia reduces neuronal damage after asphyxial cardiac arrest in rats	8.8	95	4.1	Critical Care Medicine	Hickey, RW	Hickey, RW	2000
85	Combination of systemic hypothermia and n-acetylcysteine attenuates hypoxic-ischemic brain injury in neonatal rats	3.6	94	5.5	Pediatric Research	Jatana, M	Jenkins, D	2006
86	Mild intraischemic hypothermia suppresses consumption of endogenous antioxidants after temporary focal ischemia in rats	2.9	94	3.2	Brain Research	Karibe, H	Philip R. Weinstein	1994
87	Therapeutic time window of post-ischemic mild hypothermia and the gene expression associated with the neuroprotection in rat focal cerebral ischemia	2.9	94	5.9	Neuroscience Research	Ohta, Hiroyuki	Shintani, Y	2007
88	Hypothetical pathophysiology of acute encephalopathy and encephalitis related to influenza virus infection and hypothermia therapy	1.4	93	4.0	Pediatrics International	Yokota, S	Yokota, S	2000
89	Effect of delayed MK-801 (dizocilpine) treatment with or without immediate postischemic hypothermia on chronic neuronal survival after global forebrain ischemia in rats	6.3	93	3.3	Journal of Cerebral Blood Flow and Metabolism	Dietrich, WD	Dietrich, WD	1995
90	Mild postischemic hypothermia limits cerebral injury following transient focal ischemia in rat neocortex	2.9	93	3.4	Brain Research	Yanamoto, H	Kevin S. Lee	1996
91	Regional alterations of protein-kinase-c activity following transient cerebral-ischemia—effects of intraischemic brain temperature modulation	4.7	89	3.1	Journal Of Neurochemistry	Busto, R	Busto, R	1994
92	Treatment window for hypothermia in brain injury	4.1	88	4.0	Journal of Neurosurgery	Markgraf, CG	Markgraf, CG	2001
93	Behavioral protection by moderate hypothermia initiated after experimental traumatic brain injury	4.2	88	2.9	Journal of Neurotrauma	Lyeth, BG	Lyeth, BG	1993
94	Novel thyroxine derivatives, thyronamine and 3-iodothyronamine, induce transient hypothermia and marked neuroprotection against stroke injury	8.3	88	5.5	Stroke	Doyle, Kristian P	Stenzel-Poore, MP	2007
95	Mild to moderate hypothermia prevents microvascular basal lamina antigen loss in experimental focal cerebral ischemia	8.3	88	4.6	Stroke	Hamann, GF	Hamann, GF	2004
96	Diminished neuronal damage in the rat brain by late treatment with the antipyretic drug dipyrone or cooling following cerebral ischemia	12.7	88	3.3	Acta Neuropathologica	Coimbra, C	Coimbra, C	1996
97	Therapeutic hypothermia alters microrna responses to traumatic brain injury in rats	6.3	87	7.3	Journal of Cerebral Blood Flow and Metabolism	Truettner, Jessie S	Dietrich, WD	2011
98	Role of hypothermia in the mechanism of protection against serotonergic toxicity. I. Experiments using 3,4-methylenedioxymethamphetamine, dizocilpine, CGS 19755 and NBQX	3.5	87	3.1	Journal of Pharmacology and Experimental Therapeutics	Farfel, GM	Lewis Seiden	1995
99	The relationship between intelligence and duration of circulatory arrest with deep hypothermia	6	87	3.1	Journal of Thoracic and Cardiovascular Surgery	Oates, RK	Oates, RK	1995
100	Delayed onset of prolonged hypothermia improves outcome after intracerebral hemorrhage in rats	6.3	86	4.5	Journal of Cerebral Blood Flow and Metabolism	MacLellan, CL	Colbourne, F	2004

JCRIF, Journal Citation Reports Impact Factor; average citations, average number of citations per year since publication.

The citation frequency of these 100 studies ranged from 86 to 470 times (mean = 156), with the average annual citation volume ranging from 2.9 to 19.4 times (mean = 7.3). The median publication year for these articles is 1999. Approximately one-fifth of the articles (*n* = 18) were cited more than 200 times, and only six articles were cited more than 400 times. The most-cited article, titled “Glutamate release and free radical production following brain injury: effects of posttraumatic hypothermia” was published by Globus et al. (14) in the *Journal of Neurochemistry* in 1995 and has 470 citations to date.

**Figure 2:**
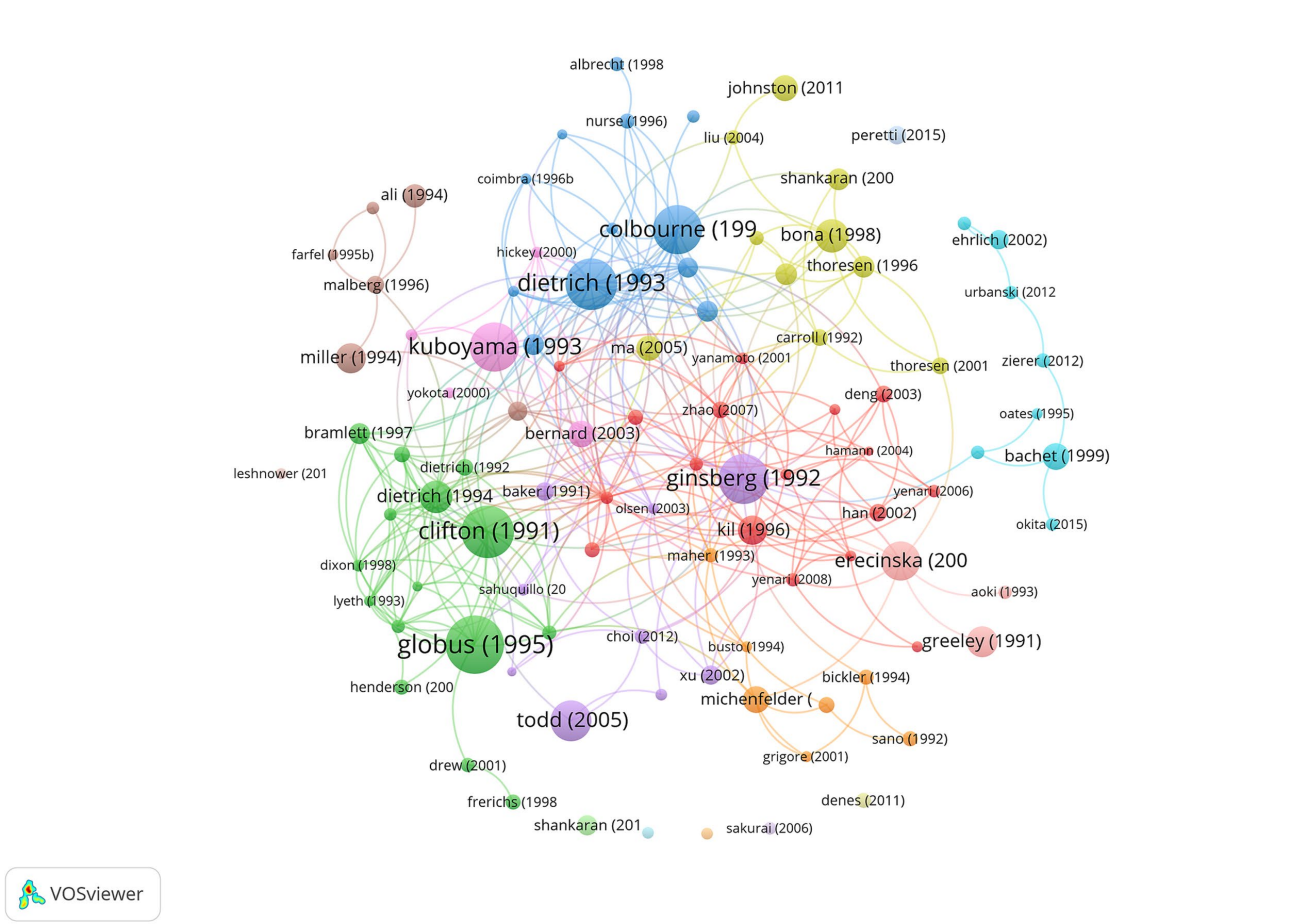
Citation of documents in hypothermic brain protection journals created using VOSviewer. Labels and circles were used to represent articles, representing authors’ name and year of publication indicated. The size of each label and circle was based on the weight of the article.

We also created a network visualization based on the number of citations per article (Figure 2). Globus et al. (14), Clifton et al. (15), Kuboyama et al. (16), Ginsberg et al. (17), Dietrich et al. (18), and Colbourne et al. (19) all received more than 400 citations, making them the largest nodes in the graph. Todd et al. (20) and Erecinska et al. (21) also received a high number of citations.

### Years and types of publications

**Figure 3:**
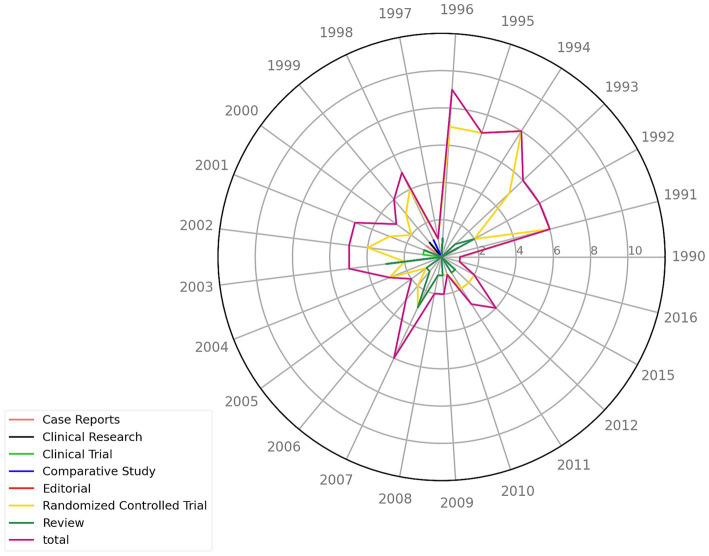
The 100 most-cited articles by publication year. The radar chart depicts the publication years of articles, spanning from 1990 to 2016. Each colored line represents a different type of article, with the outermost purple line representing the total number of publications per year.

Among the 100 most-cited articles, 1991–1996 had the most publications (*n* = 42) (Figure 3), with the highest number of publications occurring in 1996 (*n* = 9) and a smaller peak occurring in 2007 (*n* = 6). The lowest numbers of publications occurred in 1990, 1997, 2010, and 2016 (*n* = 1). Among the types of published articles, randomized controlled trials were the most common (*n* = 73), followed by reviews (*n* = 18), whereas case reports, clinical studies, and other types of articles were the least common (*n* = 9) (Table 2).

**Table 2:** Statistics on the types of studies in the 100 most-cited articles.

Study type	Count
Randomized Controlled Trial	73
Review	18
Clinical Trial	3
Comparative Study	3
Clinical Research	1
Case Reports	1
Editorial	1
Total count	100

### Contributing authors

**Figure 4:**
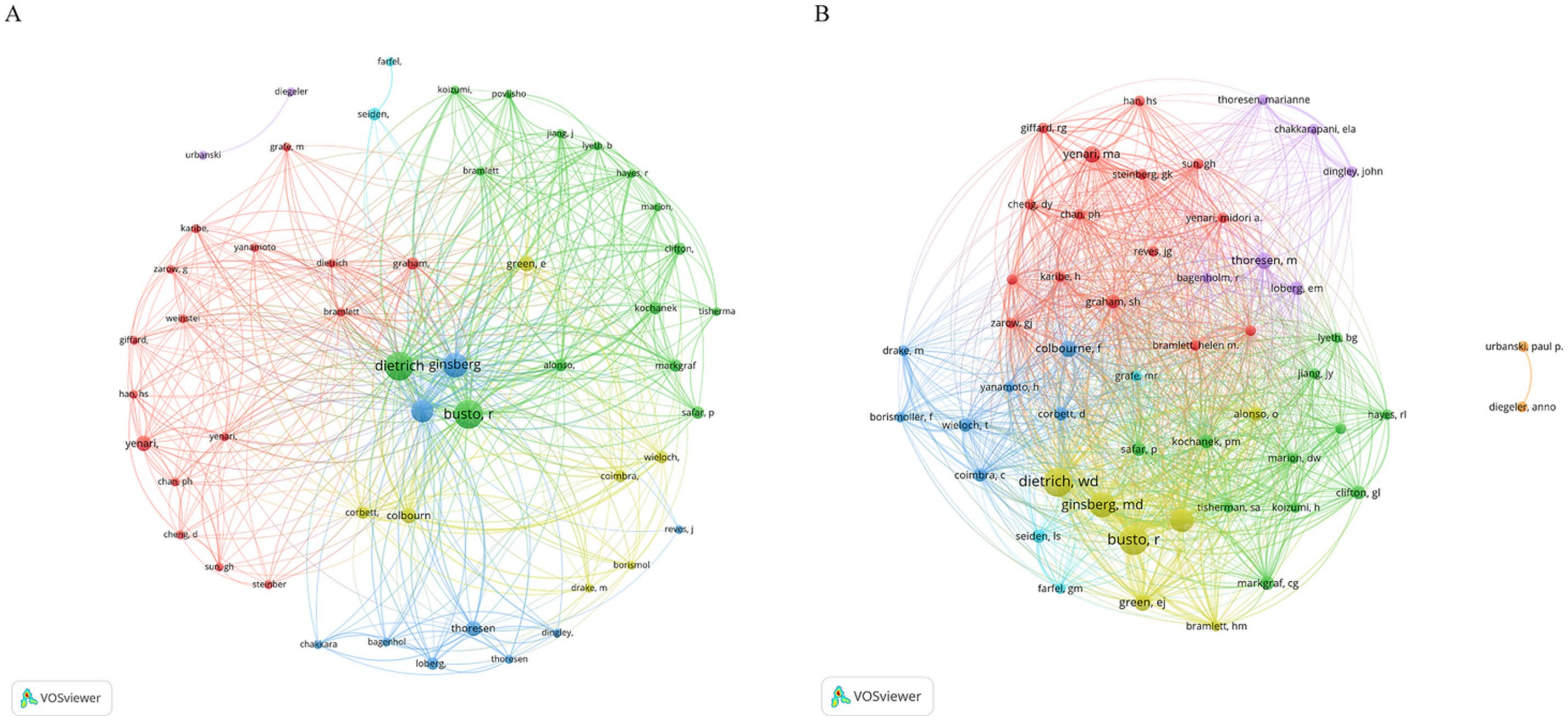
The author network visualized using VOSviewer software. The minimum number of documents by any one author is ≥2. Author citation (A) and the bibliometric coupling (B) are shown using labels and circles, showcasing each author’s name. The size of the label and circle varied according to the author’s weight.

We established a collaborative network based on the authors who published two or more papers (Figure 4). Dietrich, Ginsberg, and Busto published the most relevant articles; therefore, their nodes were the largest. In addition, we observed close collaboration among multiple authors. For example, Dietrich closely cooperated with Busto, Alonso, and others (Figure 4A). More detailed and specific collaborations between the authors can be found in the author coupling diagram (Figure 4B). There are five main color classifications, with yellow representing authors such as Dietrich, Busto, and Ginsberg, who have the highest collaborations. There are many collaborations among the authors, represented in green, such as Markgraf, Clifton, and Marion. The authors represented in red, such as Yenari, Steinberg, Chan, and Graham, strongly cooperated with each other. The authors represented in blue, such as Colbourne, Wieloch, Corbett, and Yanamoto, strongly cooperated with each other. Purple represents authors such as Thoresen, Loberg, and Chakkarapani, who participated in many collaborations.

### Contributing countries and institutions

**Figure 5:**
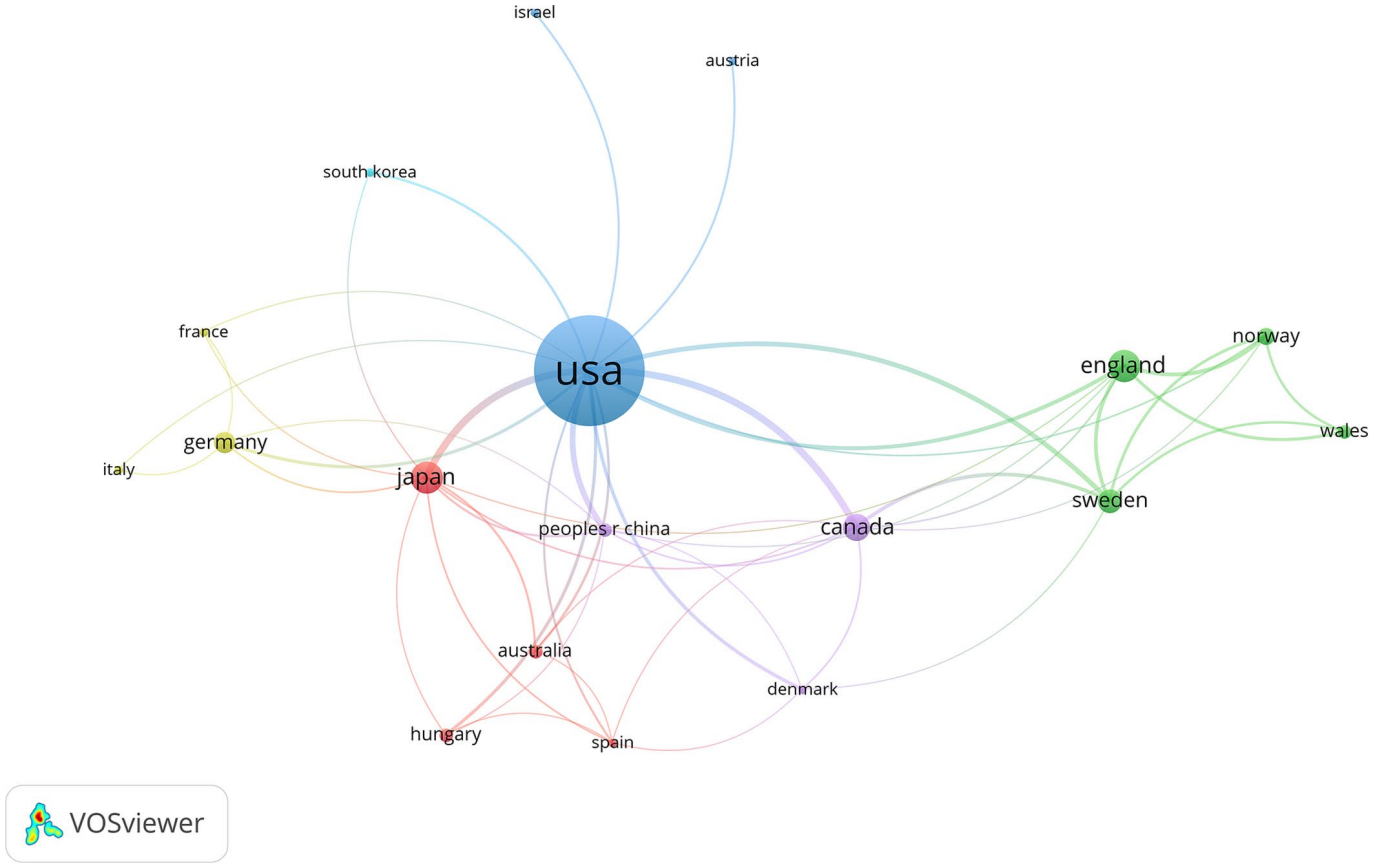
Countries cited in the 100 most-cited articles on hypothermic brain protection created by VOSviewer. The minimum number of documents from any one country is ≥1. Countries are represented by labels and circles, with the size of each country’s label and circle determined by its weight.

Visual networks and statistical charts were developed for both countries and their institutions. The 100 most-cited articles included 136 countries/regions and 258 institutions. The maps clearly indicated the presence of clusters. It is evident from the number of publications and international cooperation that the United States has the greatest influence among many countries. In addition, Japan has established strong scientific relationships with countries such as Germany, Canada, and Sweden (Figure 5). Among the countries that published articles, the United States (*n* = 51), the United Kingdom (*n* = 8), and Japan (*n* = 8) had the highest number of published articles, whereas the remaining articles were scattered among other countries (Table 3).

**Table 3:** Countries of the 100 articles in hypothermic brain protection journals.

Journal	Country	Year	Count
Journal of Cerebral Blood Flow and Metabolism	United States	1981	15
Stroke	United States	1970	10
Anesthesiology	United States	1940	5
Journal of Neurotrauma	United States	1984	5
Journal of Thoracic and Cardiovascular Surgery	United States	1936	5
Acta Neuropathologica	Germany	1961	4
Annals of Thoracic Surgery	United States	1965	4
Brain Research	Netherlands	1966	4
Critical Care Medicine	United States	1973	4
Journal of Neurosurgery	United States	1944	4
Journal of Pharmacology and Experimental Therapeutics	United States	1909	4
Experimental Neurology	United States	1959	3
Pediatric Research	United States	1967	3
Archives of Disease in Childhood-Fetal and Neonatal Edition	United Kingdom	1996	2
Cerebrovascular and Brain Metabolism Reviews	United States	1989	2
European Journal of Cardio-Thoracic Surgery	Netherlands	1987	2
Journal of Neurochemistry	United States	1956	2
Journal of Neuroscience	United States	1981	2
Lancet Neurology	United Kingdom	2002	2

**Figure 6:**
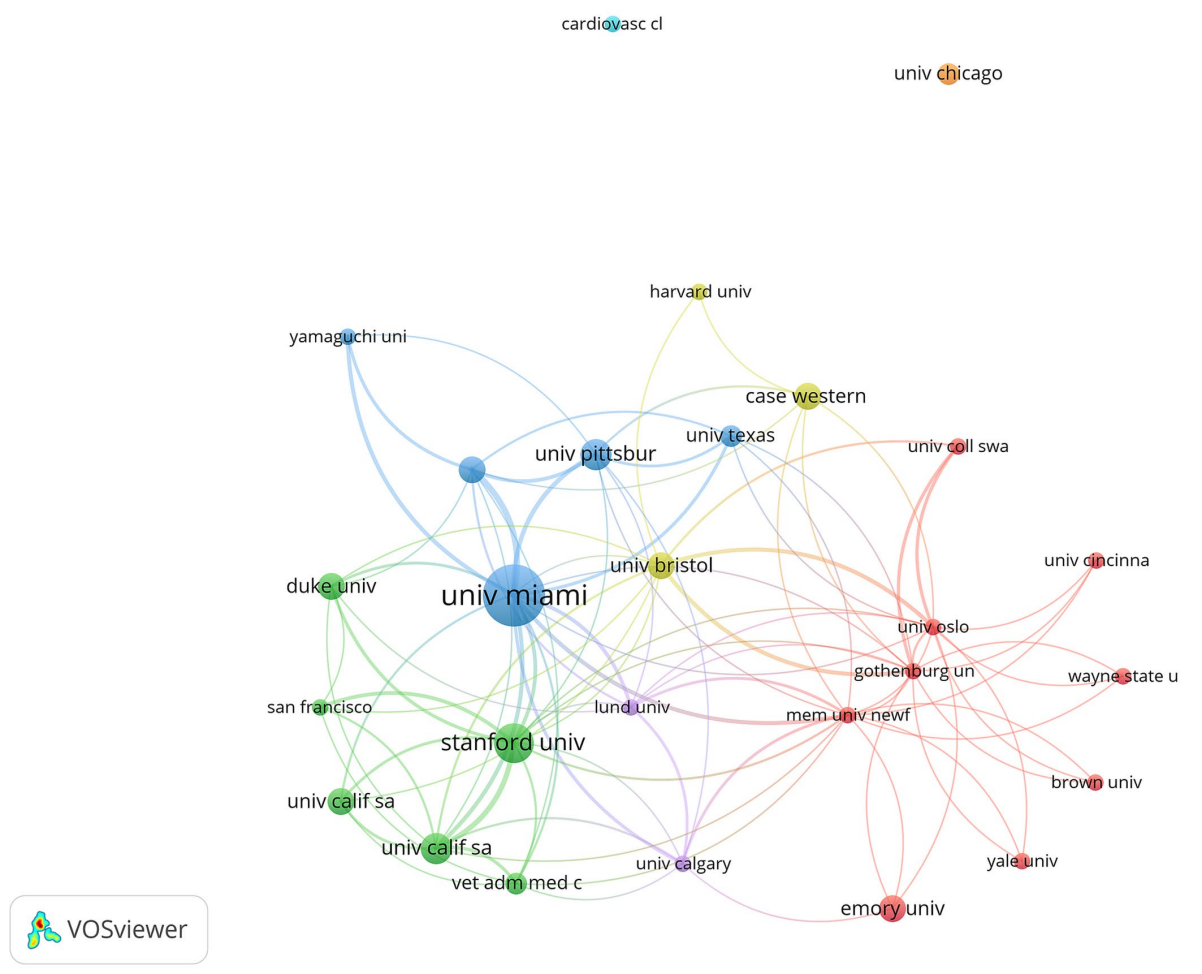
Citation of institutions in the 100 most-cited articles in hypothermic brain protection journals created by VOSviewer. The minimum number of documents from any one country is ≥2. Institutions are represented by labels and circles, with the size of each institution’s label and circle determined by its weight.

Multiple institutions also formed regional cooperative networks. The University of Miami has close cooperation with the University of Texas, the University of Pittsburgh, and others. Stanford University has close cooperation with Duke University, the University of California, San Francisco University, and others. The Memorial University of Newfoundland cooperates closely with Emory University, Yale University, and others (Figure 6).

### Journal of publication

Journals with at least two publications and their main characteristics are listed in Table 4. There are 19 journals on the list, and the two journals with the highest publication volumes in the field of hypothermic brain protection are the *Journal of Cerebral Blood Flow and Metabolism* and *Stroke*, with 15 and 10 articles published, respectively. Additionally, five articles were published in *Anesthesiology*, the *Journal of Neurotrauma*, and the *Journal of Thoracic and Cardiovascular Surgery*.

**Table 4:** Journal distribution and main characteristics of the 100 most-cited articles on hypothermic brain protection.

Country	Count
USA	51
Japan	8
UK	8
Canada	6
Sweden	5
Germany	4
Norway	3
Australia	2
China	2
Wales	2
Hungary	2
Italy	1
Israel	1
France	1
Austria	1
Denmark	1
South Korea	1
SPAIN	1
Total count	100

The minimum number of articles from any one journal is ≥2. SJR, SCImago Journal Rank 2022; cites/doc: total citations/total documents; Year: journal establishment year.

### Research topics

**Figure 7:**
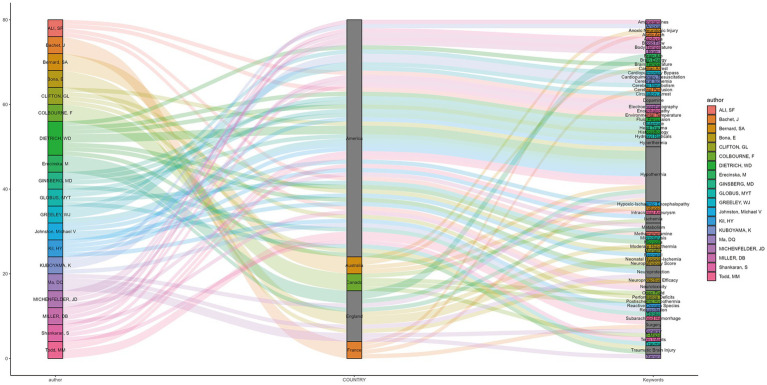
Sankey plot showing the relationships among authors, countries, and research keywords of the 20 most-cited articles. The left column represents the authors’ name, the middle column represents the authors’ country, and the right column represents the articles’ keywords. These columns are connected with corresponding lines on a one-to-one basis.

The relationships among the authors, countries, and research keywords of the 20 most-cited articles in hypothermic brain protection journals are shown in Figure 7. Other keywords related to hypothermic brain protection with a high frequency of occurrence included hyperthermia, dopamine, ischemia, metabolism, surgery, and trauma.

## Discussion

With continuous advances in science and technology, research in the biomedical field is expanding in terms of breadth and depth, leading to an exponential increase in the number of related studies (113). Effectively screening useful information from this vast body of literature poses a major challenge for researchers. Bibliometric analysis, a key tool in modern medical research, allows researchers to quantitatively analyze scientific literature to reveal development dynamics and trends in the research field. Using bibliometric analysis, researchers can better understand the development of a discipline, identify cutting-edge fields and research hotspots, evaluate the impact and quality of academic achievements, and provide valuable references and guidance for future medical research and strategic planning (10).

Hypothermic brain protection is a key area of medical research aimed at reducing brain injury and improving the tolerance to cerebral ischemia, trauma, and other conditions. Hypothermic brain protection techniques have shown potential neuroprotective effects in patients with neurological ischemia or injury (114–116). Although there are challenges to its clinical application, research on hypothermic brain protection is advancing, providing new ideas and methods for improving the treatment of brain injuries and neurological diseases.

The research that is most cited in a specific field is often considered a milestone and can be referred to as “classic (117, 118).” The frequency of citations in a paper generally reflects its importance, indicating that it has gained recognition from researchers in the relevant field as well as sparking discussions and guiding new research directions (119, 120). Owing to its pioneering contributions, this study provides important reference values for further analysis. This study identified and analyzed the 100 most-cited articles in the field of hypothermic brain protection, providing an historical overview of the development of the research field over time, defining interesting trends, and potentially offering clues for the future development of basic research and clinical practice in hypothermic brain protection.

Since Busto et al. (121, 122) first proposed the use of mild hypothermia (33–35°C) to treat brain injuries in the 1980s, experts have recognized its protective effects on the body, particularly on the brain. The question of whether lowering body temperature to induce hypothermia can effectively shield the brain has sparked interest in clinical and basic research. The 100 most cited articles listed in this study have shown that hypothermia can improve brain function and provide significant protection (1, 14–112). The current mechanisms of hypothermic brain protection include reducing brain energy metabolism, protecting the blood brain barrier, decreasing brain swelling and pressure, preventing lactic acid accumulation, limiting the release of harmful amino acids, blocking the detrimental effects of calcium, inhibiting nitric oxide production, reducing the generation of oxygen radicals, enhancing the elimination of oxygen radicals, suppressing the expression of genes associated with cellular damage, and reducing inflammation and neuronal cell death (2, 123).

Hypothermic brain protection is used to lower a patient’s body or brain temperature to decrease brain oxygen consumption and facilitate recovery (124). Mild hypothermia (33–35°C) and moderate hypothermia (28–32°C) are commonly used, and studies have indicated that 33°C is the optimal temperature for treatment (125). Deep hypothermia (17–27°C) is reserved for specific patients (for example, those with aortic stenosis or aortic dissection) due to more severe complications (126).

However, some recent large-scale clinical studies have refuted this view. In a study conducted in 2005 to determine whether hypothermia during craniotomy can improve the prognosis of patients with acute aneurysmal subarachnoid hemorrhage, there was no significant difference in hospitalization time, total hospitalization time, or follow-up mortality in the intensive care unit between the hypothermia group and the normal temperature group during craniotomy (20). Another randomized controlled trial published in 2010 showed no correlation between intraoperative hypothermia or supplementation of protective drugs and neurological prognosis in patients undergoing temporary clipping during cerebral aneurysm surgery (127). At the same time, some systematic reviews also expound similar viewpoints (128–130).

There is a guideline stating that the duration of brain hypothermia should be sufficient to provide brain protection (131). For patients with craniocerebral injury, it is challenging to achieve favorable clinical outcomes with short-term (24–48 h) mild hypothermia treatment. It is recommended that the duration of mild hypothermia treatment for such patients be maintained for at least 3–5 days. Therefore, additional research is required to determine the appropriateness of utilizing low temperatures in varying circumstances, along with the corresponding low-temperature strategy and duration of maintenance.

The most-cited studies in the field of hypothermic brain protection generally describe the effects of posttraumatic hypothermia on neuronal damage in rats with traumatic brain injury (TBI) (14). Research has shown that TBI leads to a significant increase in the glutamate and hydroxyl radical levels in the brain, with a positive correlation between these two factors. Post-traumatic hypothermia effectively suppresses these elevations, indicating a potential link between glutamate release and hydroxyl radical production in the brain after TBI. This groundbreaking discovery has had a significant guiding influence on clinical treatment, making it the most influential article. Six articles, with a total citation count of over 400 in this field, can be referred to as “classics.” The six authors and their teams—Globus et al. (14), Clifton et al. (15), Kuboyama et al. (16), Ginsberg et al. (17), Dietrich et al. (18), and Colbourne et al. (19)—have all made significant contributions to further research in the field of hypothermic brain protection.

The temporal distribution of these 100 articles revealed that they were published between 1990 and 2016, whereas the years that produced a relatively large number of highly influential articles were 1991–1996 and 2007. Furthermore, it must be emphasized that the total citation count of publications over the past 3 years may have been underestimated, considering that recently-published articles will take time to attract citations. Over time, an increasing number of recently-published studies have become highly cited (132). Among the 100 articles, the proportion of basic research was the highest (*n* = 73), followed by reviews (*n* = 18), whereas the proportions of clinical research and case reports were low (*n* = 8). These findings indicate that although we have observed the protective effect of hypothermia, we still have only a partial understanding of its mechanism, and the study of the specific mechanism of hypothermic brain protection remains a hot topic (123).

Regarding countries and institutions, most of the 100 most-cited articles in the field of hypothermic brain protection were from the United States (*n* = 51), which also had an overwhelming number of citations, indicating that the United States is the most influential country in this field. The United States has always led the world in the field of hypothermic brain protection research, and its continuously innovative medical technology and cutting-edge research results have inspired tremendous progress. The most-cited authors were from the United States. The University of Miami and Stanford University the institutions with the most cited articles, reflecting their authority in the field of hypothermic brain protection (35, 39). In terms of international cooperation, the United States cooperates closely with multiple countries, whereas Japan cooperates closely with Germany, the United Kingdom, and Sweden. At the institutional level, Stanford University cooperates closely with the University of California, Emory University, and Duke University (42).

The most-cited research in this field is more likely to be published in highly-influential neurosurgical journals such as the *Journal of Cerebral Blood Flow and Metabolism and Stroke* (15, 36). In addition, several studies published in these journals, apart from those in the field of neurosurgery, involve the intersection of anesthesia and critical care, as well as pediatrics and neurosurgery, such as anesthesiology, critical care medicine, and pediatric research (16, 22, 30). Interestingly, these results suggest that hypothermic brain protection has aroused great interest not only among neurologists but also among anesthesiologists and pediatricians due to its close connection to their clinical work.

The Sankey plot illustrated the relationships among authors, countries, and agreed-upon keywords. The keywords used were further extended to include “hypothermia,” “brain,” “neuroprotection,” “surgery,” “trauma,” “ischemia,” “metabolism,” and others. A relatively novel keyword, “xenon,” was used to explore the treatment of neonatal hypoxic-ischemic brain injury (31, 38, 73). The experimental data from these studies showed that low-concentration xenon combined with mild hypothermia may be a safe and effective treatment for perinatal asphyxia. Most articles mentioned keywords such as “hypothermia” and “neuroprotection” in their titles; however, a few articles do not mention them. We recommend that the terms “hypothermia” and “neuroprotection” are included in the title so readers can easily identify the nature of the article and index it in search databases.

Keywords Plus^®^ was used with the Clarivate Analytics^1^ algorithm, which is based on repeated words or phrases appearing in the reference lists of indexed articles (133). In the absence of author keywords, Keywords Plus^®^ is considered to have special value. However, compared with keywords, the number of Keywords Plus^®^ was greater, and the concentration of keywords was not strong. The use of Keyword Plus^®^ to construct Sankey plots may have caused data distortion. Therefore, we constructed the Sankey plot using keywords. Seven of the 20 most-cited articles in this field did not provide keywords, and three researchers summarized the keywords after their discussion according to Keywords Plus^®^.

This study has several limitations. The first limitation is inherent in citation analysis which is based on the absolute number of citations of an article. The number of citations is a substitute for this influence; however many factors can affect the citation rate. Generally the number of citations an article receives reflects its impact and level of recognition by academic and clinical communities. However the number of citations depends on many factors such as factors related to the paper including quality length publication year and literature type; factors related to journals such as journal influence language and publication format; and factors related to the author such as reputation academic ranking and productivity. Second only English versions of the articles were included in the study; however English is the most widely-used language worldwide making it possible for articles published in English to be widely read and cited. Third the search was conducted only on the WOS database which may have resulted in missing several relevant publications from other databases such as Google Scholar Scopus and PubMed. Searching databases from multiple sources can yield more complete citation results because the number of citations varies among databases from different sources (134). However different databases have different reference-counting methods which may be unsuitable for merging data from different databases. Among these databases WOS is one of the most commonly used databases for analyzing highly-cited articles in a particular field (135–137). The fourth limitation was the time factor because the WOS currently covers only articles published after 1980. We may have missed articles published before 1980 with higher citation frequencies than those included in this study. Additionally recently published articles require time to attract citations. Therefore the citation frequency in early studies should be higher than that in recently published studies and some recently-published representative works may not be included. Quotations may not fully represent the true academic value and the impact of a study should be evaluated from all aspects. Future work can start by updating the time periods searching in and merging multiple databases and establishing algorithms for the influence of new and old articles. Nevertheless the results of this study provide valuable insights for researchers.

## Conclusion

This bibliometric study offers a comprehensive overview of the advancements, trends, and current trajectories in both basic research and clinical applications within the domain of hypothermic brain protection. By conducting a bibliometric analysis of the WOS database, this study identified and thoroughly analyzed the 100 most impactful articles that have significantly contributed to advancing this field. Rapid growth has been observed in the field of hypothermic brain protection. The United States emerged as a dominant force in terms of the number of highly influential articles, prominent academic institutions, and leading scientists in this area of research. Predominantly, articles in this field focus on basic research and delve into the underlying mechanisms. Keywords, such as “hypothermia” and “neuroprotection” intersect with other fields, enriching the comprehension of mechanisms and broadening their applicability. Continuous and profound research endeavors in this sphere promise a deeper understanding of the molecular pathophysiological mechanisms underlying various diseases, thereby unveiling potential therapeutic targets.

## Data Availability

The original contributions presented in the study are included in the article/Supplementary material, further inquiries can be directed to the corresponding authors.

## Footnotes

^1^ https://clarivate.com/
