# Supplementary material for: The 100 most-cited articles in hypothermic brain protection journals: a bibliometric and visualized analysis
Source: Front Neurol. 2024 Nov 5;15:1433025. doi: 10.3389/fneur.2024.1433025 (PMC11575058; doi:10.3389/fneur.2024.1433025)
Supplement: Supplementary file 1 [file Table_1.DOCX]

Web of Science [1980-present]

1. search strategy：

#1 ((((ALL=(hypothermia)) OR ALL=(Hypothermias)) OR ALL=(Hypothermia, Accidental)) OR ALL=(Accidental Hypothermia)) OR ALL=(Accidental Hypothermias)

#2 ((((((((((ALL=(Central Nervous System)) OR ALL=(Central Nervous Systems)) OR ALL=(Nervous System, Central)) OR ALL=(Nervous Systems, Central)) OR ALL=(Systems, Central Nervous)) OR ALL=(Cerebrospinal Axis)) OR ALL=(Axi, Cerebrospinal)) OR ALL=(Axis, Cerebrospinal)) OR ALL=(Cerebrospinal Axi)) OR ALL=(Brain)) OR ALL=(Encephalon)

#3 (ALL=(protect)) OR ALL=(protection)

#4 #1 AND #2 AND #3

2.Result：

1847
