# Supplementary material for: The 100 most-cited articles in hypothermic brain protection journals: a bibliometric and visualized analysis
Source: Front Neurol. 2024 Nov 5;15:1433025. doi: 10.3389/fneur.2024.1433025 (PMC11575058; doi:10.3389/fneur.2024.1433025)
Supplement: Supplementary file 2 [file Table_2.DOCX]

1.Python (version 3.11.5) and it’s packges:

pandas (version 2.1.0)、rpy2 (version 3.5.14)、chardet (version 5.2.0)、matplotlib (version 3.7.2)

2.R (version 4.3.1) and it’s packges:

Ggplot (version 2 3.4.3)、ggalluvial (version 0.12.5)
